# Separating metagenomic short reads into genomes via clustering

**DOI:** 10.1186/1748-7188-7-27

**Published:** 2012-09-26

**Authors:** Olga Tanaseichuk, James Borneman, Tao Jiang

**Affiliations:** 1 Department of Computer Science and Engineering, University of California, Riverside, CA, USA; 2 Department of Plant Pathology and Microbiology, University of California, Riverside, CA, USA

**Keywords:** Metagenomics, NGS short reads, Genome separation, Clustering

## Abstract

**Background:**

The metagenomics approach allows the simultaneous sequencing of all genomes in an environmental sample. This results in high complexity datasets, where in addition to repeats and sequencing errors, the number of genomes and their abundance ratios are unknown. Recently developed next-generation sequencing (NGS) technologies significantly improve the sequencing efficiency and cost. On the other hand, they result in shorter reads, which makes the separation of reads from different species harder. Among the existing computational tools for metagenomic analysis, there are similarity-based methods that use reference databases to align reads and composition-based methods that use composition patterns (*i.e.*, frequencies of short words or *l*-mers) to cluster reads. Similarity-based methods are unable to classify reads from unknown species without close references (which constitute the majority of reads). Since composition patterns are preserved only in significantly large fragments, composition-based tools cannot be used for very short reads, which becomes a significant limitation with the development of NGS. A recently proposed algorithm, AbundanceBin, introduced another method that bins reads based on predicted abundances of the genomes sequenced. However, it does not separate reads from genomes of similar abundance levels.

**Results:**

In this work, we present a two-phase heuristic algorithm for separating short paired-end reads from different genomes in a metagenomic dataset. We use the observation that most of the *l*-mers belong to unique genomes when *l* is sufficiently large. The first phase of the algorithm results in clusters of *l*-mers each of which belongs to one genome. During the second phase, clusters are merged based on *l*-mer repeat information. These final clusters are used to assign reads. The algorithm could handle very short reads and sequencing errors. It is initially designed for genomes with similar abundance levels and then extended to handle arbitrary abundance ratios. The software can be download for free at http://www.cs.ucr.edu/∼tanaseio/toss.htm.

**Conclusions:**

Our tests on a large number of simulated metagenomic datasets concerning species at various phylogenetic distances demonstrate that genomes can be separated if the number of common repeats is smaller than the number of genome-specific repeats. For such genomes, our method can separate NGS reads with a high precision and sensitivity.

## Background

Metagenomics [1] is a new field of study that provides a deeper insight into the microbial world compared to the traditional single-genome sequencing technologies. Traditional methods for studying individual genomes are well developed. However, they are not appropriate for studying microbial samples from the environment because traditional methods rely upon cultivated clonal cultures while more than 99% of bacteria are unknown and cannot be cultivated and isolated [2]. Metagenomics uses technologies that sequence uncultured bacterial genomes in an environmental sample directly [3], and thus makes it possible to study organisms which cannot be isolated or are difficult to grow in a lab. It provides hope for a better understanding of natural microbial diversity as well as their roles and interactions. It also opens new opportunities for medicine, biotechnology, agricultural studies and ecology.

Many well-known metagenomics projects use the whole genome shotgun sequencing approach in combination with Sanger sequencing technologies. This approach has produced datasets from the Sargasso Sea [4], Human Gut Microbiome [5] and Acid Mine Drainage Biofilm [6]. However, new sequencing technologies have evolved over the past few years. The sequencing process has been greatly parallelized, producing millions of reads with much faster speed and lower cost. Since NGS technologies are much cheaper, they allow sequencing to be performed at a much greater depth. The only drawback is that read length is reduced - NGS reads are usually of lengths 25-150 (Illumina/SOLiD) compared to 800-1000 bps in Sanger reads.

The primary goals of metagenomics are to describe the populations of microorganisms and to identify their roles in the environment. Ideally, we want to identify complete genomic sequences of all organisms present in a sample. However, metagenomic data is very complex, containing a large number of sequence reads from many species. The number of species and their abundance levels are unknown. The assembly of a single genome is already a difficult problem, complicated by repeats and sequencing errors which may lead to high fragmentation of contigs and misassembly. In a metagenomic data, in addition to repeats within individual genomes, genomes of closely related species may also share homologous sequences, which could lead to even more complex repeat patterns that are very difficult to resolve. A lot of research has been done for assembling single genomes [7-10]. But due to the lack of research on metagenomic assemblers, assemblers designed for individual genomes are routinely used in metagenomic projects [4,6]. It has been shown that these assemblers may lead not only to misassembly, but also severe fragmentation of contigs [11]. A plausible approach to improve the performance of such assemblers is to separate reads from different organisms present in a dataset before the assembly.

Many computational tools have been developed for separating reads from different species or groups of related species (we will refer to the problem as the clustering of reads). Some of the tools also estimate the abundance levels and genome sizes of species. These tools are usually classified as similarity-based (or phylogeny-based) and composition-based. The purpose of similarity-based methods is to analyze the taxonomic content of a sample. Small-scale approaches involving 16S rRNAs and 18S rRNAs [12] are commonly used to determine evolutionary relationships by analyzing fragments that contain marker genes and comparing them with known marker genes. These methods take advantage of small number of fragments containing marker genes and require reads to have at least 1000 bps. Two other tools handle a larger number of fragments: MEGAN [13] and CARMA [14]. MEGAN aligns reads to databases of known sequences using BLAST [15] and assigns reads to taxa by the lowest common ancestor approach. CARMA performs phylogenetic classification of unassembled reads using all Pfam domains and protein families as phylogenetic markers. These two methods work for very short reads (as short as 35 bps for MEGAN and 80 bps for CARMA). However, a large fraction of sequences may remain unclassified by these methods because of the absence of closely related sequences in the databases.

The second class of methods use compositional properties of the fragments (or reads). These methods are based on the fact that some composition properties, such as CG content and oligonucleotide frequencies are preserved across sufficiently long fragments of the same genome, and vary significantly between fragments from different organisms. *K*-mer frequency is the most widely used characteristics for binning. For example, the method in [16] utilizes the property that each genome has a stable distribution of *k*-mer frequencies for *k* = 1.6 in fragments as short as 1000 bps. It shows that these fragments have very similar “barcodes” and thus can be clustered based on their barcode similarities. Barcode similarity also correlates with phylogenetic closeness between genomes. The main challenge in the *k*-mer frequency approach is that these frequencies produce large feature vectors, which can be even larger than the sizes of fragments. Different methods have been proposed to deal with this problem. CompostBin [17], which uses hexamer frequencies, adopts a modified principle component analysis to extract the top three meaningful components and then cluster the reads based on principal component values. Self-organizing maps are another way to reduce dimensionality by mapping multidimensional data to two dimensional space. The work in [18] uses SOMs for tri- and tetranucleotide frequency vectors. In TETRA [19], z-scores are computed for tetranucleotide frequencies and fragments are classified by the Pearson correlation of their z-scores. MetaCluster 3.0 [20] uses Spearman Footrule distance between *k*-mer feature vectors. Another composition feature is used in TACOA [21]: the ratio between observed oligonucleotide frequencies and expected frequencies given the CG content. To cluster fragments, the *k*-NN approach is combined with the Gaussian kernel function. The main limitation of composition based methods is that the length of fragments may significantly influence their performance. In general, these methods are not suitable for fragments shorter than 1000 bps [22].

AbundanceBin [23] is a recently developed tool for binning reads that uses an approach different from the above similarity and composition based techniques. It is designed to separate reads from genomes that have different abundance levels. It computes frequencies of all *l*-mers in a metagenomic dataset and, assuming that these frequencies come from a mixture of Poisson distributions, predicts the abundance levels of genomes and clusters *l*-mers according to their frequencies. Then reads are clustered based on the frequencies of their *l*-mers. This method is suitable for very short NGS reads. The limitation is that genomes whose abundance levels do not differ very much (within ratio 1:2) will not be separated.

In this paper, we present a two-phase heuristic algorithm for separating short paired-end reads from different organisms in a metagenomic dataset, called TOSS (*i.e.*, TOol for Separating Short reads). The basic algorithm is developed to separate genomes with similar abundance levels. It is based on several interesting observations about unique and repeated *l*-mers in a metagenomic dataset, which enables us to separate unique *l*-mers (each of which belongs to only one genome and is not repeated) from repeats (*l*-mers which are repeated in one or more genomes) at the beginning of the first phase of the algorithm. During the first phase, unique *l*-mers are clustered so that each cluster consists of *l*-mers from only one of the genomes. This is possible due to the observation that most *l*-mers are unique within a genome and, moreover, within a metagenomic dataset. During the second phase, we find connections between clusters through repeated regions and then merge clusters of *l*-mers that are likely to belong to the same organism. Finally, reads are assigned to clusters. In order to handle metagenomic datasets with genomes of arbitrary abundance ratios, we combine the method with AbundanceBin which attempts to separate *l*-mers from genomes with significantly different abundance levels. The integrated method works for very short reads, and is able to handle multiple genomes with arbitrary abundance levels and sequencing errors. We test the method on a large number of simulated metagenomic datasets for microbial species with various phylogenetic closeness according to the NCBI taxonomy [24,25] and show that genomes can be separated if the number of common repeats is less then the number of genome-specific repeats. For example, genomes of different species of the same genus often have a large number of common repeats and thus are very hard to separate. In the tests, our method is able to separate fewer than a half of groups of such closely related genomes. However, with the decrease in the fraction of common repeats, the ability to accurately separate genomes significantly increases. Due to the lack of appropriate short read clustering tools for comparison, we modify a well-known genome assembly software, Velvet [26], to make it behave like a genome separation tool and compare our clustering results with those of the modified Velvet.

The paper is organized as follows. In the “Methods” section, we consider properties of *l*-mers in a metagenomic dataset and make several observations which form the intuition behind the algorithm, present the main algorithm, and extend the algorithm to handle arbitrary abundance ratios. The “Experimental results” section gives the comparison with the modified Velvet on short reads and comparison with the well-known composition-based tool CompostBin on longer reads.

## Methods

### Preliminaries

The algorithm we are going to present is based on *l*-mers from metagenomic reads. In this section, we will discuss some properties of *l*-mers that are important for our algorithm, and also make some important observations that lead to the intuition behind the algorithm.

First, let us analyze the expected number of occurrence of *l*-mers in reads sequenced from a single genome of length *G*. Let the number of paired-end reads be *N* (which corresponds to 2*N* read sequences) and read length *L*. In shotgun sequencing projects, as well as NGS, the reads are randomly distributed across the genome. Since reads may begin at any positions of the genome with equal probability, Lander and Waterman suggested that the left ends of reads follow a Poisson distribution [27], which means that the probability for a read to begin at a given position of the genome is *α* = 2*N*/(*G* − *L* + 1) and the number of reads starting at each position has a Poisson distribution with parameter *α*. Consider a substring *w*_*i*_ of length *l* that begins at the *i*-th position of the genome. Let *x*(*w*_*i*_) be the number of reads that cover this particular *l*-mer. Since there are *L* − *l* + 1 possible starting positions for such reads, *x*(*w*_*i*_) has a Poisson distribution with parameter *λ* = *α*(*L* − *l* + 1) (this parameter represents the effective coverage [27,28]). This analysis assume that the *l*-mer *w*_*i*_ occurs uniquely in the genome, but in general, an *l*-mer may occur multiple times. Suppose that an *l*-mer *w* has *n*(*w*) copies in the genome located at positions *i*_1_,…,*i*_*n*(*w*)_. Then the total number of reads containing *w* is $x(w)=\overset{n(w)}{\underset{j=1}{\sum }}x(w_{i_{j}})$. If we assume that a read covers at most one copy of *w*, then $x(w_{i_{j}}),j=1,\ldots ,n(w)$, are independent and identically distributed. So by the additivity property of the Poisson distribution, the total number of occurrences of *w* in the reads, *x*(*w*), follows a Poisson distribution with parameter *α*(*L* − *l* + 1)*n*(*w*). In [29], this model is used to find repeat families for a single genome, where a repeat family is a collection of *l*-mers that have the same number of copies in the genome.

**Figure 1:**
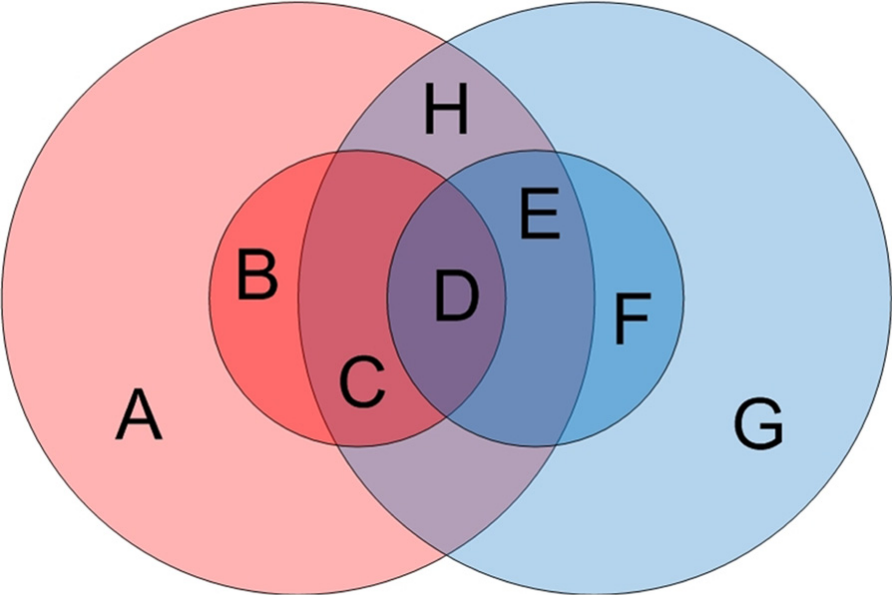
**Unique and repeated*l*-mers.A** and **G**: unique *l*-mers; **B** and **F**: individual repeats; **C**, **D**, **E**, and **H**: common repeats where **C** and **E** contain repeats only for one of the genomes, **D** contains repeats for both genomes, and **H** contains *l*-mers that are common to both genomes. Note that $n_{1}^{\mathrm{dist}}=A+B+C+D+E+H$ , $n_{2}^{\mathrm{dist}}=C+D+E+F+G+H$, $n_{1}^{\mathrm{uniq}}=A+E+H$, $n_{2}^{\mathrm{uniq}}=G+C+H$, *n*^*dist*^ = *A* + *B* + *C* + *D* + *E* + *F* + *G* + *H*and *n*^*uniq*^ = *A* + *G*.

**Figure 2:**
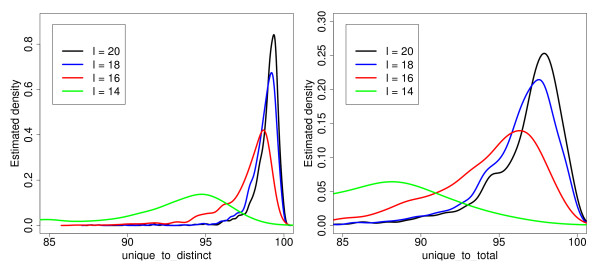
**The fraction of unique *l*-mers.** Estimated density functions of the fraction of unique *l*-mers in fully sequenced bacterial genomes for *l* = 14,16,18,20. Left: the ratio of unique *l*-mers to distinct *l*-mers. Right: the ratio of unique *l*-mers to total *l*-mers.

In a metagenome, besides repeats that occur within individual genomes, genomes of different species may share common *l*-mers. Consider *S* genomes *g*_*j*_*j*=1,…,*S*, and assume that an *l*-mer *w* has *n*_*j*_(*w*) copies in each genome *g*_*j*_, *j*=1,…,*S*. Then the number of reads containing *w* is $x(w)=\overset{S}{\underset{j=1}{\sum }}\alpha_{j}(L-l+1)n_{j}(w)=\overset{S}{\underset{j=1}{\sum }}\lambda_{j}n_{j}(w)$, where *λ*_*j*_ represents the effective coverage of genome *g*_*j*_. Since sequencing depth is the same for all genomes, we will refer to it as the abundance. This model is quite difficult to use in practice because we do not know the number of genomes and their repeat structures, common repeats and abundance levels. A simplification of this model is used in AbundanceBin [23], by assuming that for large enough *l*, most *l*-mers appear only once in the genomes (not that in AbundanceBin, 20-mers are considered, compared to 12-mers considered in [29]). This allows the authors to estimate the abundance levels of genomes by modeling the abundance levels of the genomes as a mixture of Poisson distributions, where the parameters are the abundance levels of the genomes and their observed values are the counts of the *l*-mers (*i.e.*, the number of reads containing these *l*-mers). This approach works well if the abundance levels are sufficiently different. Also, it is applicable only if the above simplifying assumption holds. Below, we will discuss the validity of this assumption in real bacterial genomes and make three important observations about the distribution of *l*-mers. Before going into the details of the observations, let us introduce some notations. Consider two different genomes, *g*_1_ and *g*_2_, of lengths *G*_1_and *G*_2_. Let $n_{1}^{\mathrm{dist}}$ denote the number of distinct *l*-mers in *g*_1_, $n_{1}^{\mathrm{uniq}}$ the number of *l*-mers that have only one copy in *g*_1_ (we will call them the unique *l*-mers in *g*_1_) and $n_{1}^{\mathrm{tot}}$ the total number of *l*-mers in *g*_1_ (including copies). Obviously $n_{1}^{\mathrm{tot}}=G_{1}-l+1$. The notations for genome *g*_2_are defined similarly (see Figure 1 for an illustration of these notations). Our first observation is the following: (1) Most of the *l*-mers in a bacterial genome are unique in this genome. To confirm it, we have computed the ratio of unique *l*-mers to distinct *l*-mers for all complete bacterial genomes downloaded from NCBI. Figure 2 shows the estimated density of this value. We can conclude that fraction of unique *l*-mers with *l* = 20 is between 96% and 100% for most of complete bacterial genomes.

**Figure 3:**
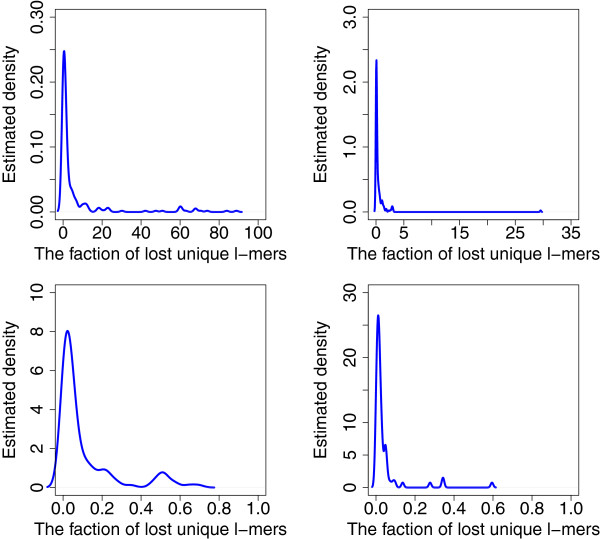
**The fraction of lost unique*l*-mers.** Estimated density of the ratio of the number of unique *l*-mers *n*^*uniq*^ to the total number of *l*-mers that are unique in an individual genome, $n_{1}^{\mathrm{uniq}}+n_{2}^{\mathrm{uniq}}$. Top left: pairs of genomes from the same genus but different species. Top right: pairs of genomes from the same family but different genera. Bottom left: pairs of genomes from the same order but different families. Bottom right: pairs of genomes from the same class but different orders.

In order to explain the second observation, let us introduce more notations. Let us consider *l*-mers from two genomes *g*_1_ and *g*_2_. Denote by *n*^*dist*^ the total number of distinct *l*-mers in both genomes together. We say that an *l*-mer is unique if it is present only in one genome and, moreover, unique in this genome. Then *n*^*uniq*^ denotes the number of unique *l*-mers in the genomes. Obviously, $n_{1}^{\mathrm{uniq}}+n_{2}^{\mathrm{uniq}}\geq n^{\mathrm{uniq}}$, because some *l*-mers that are unique in one genome may not be unique in both genomes due to common repeats. Our second observation is concerned with the percentage of unique *l*-mers in a metagenome: (2) Most *l*-mers are unique in a metagenome if it consists of genomes of species separated by sufficiently large phylogenetic distances. To validate it, we show that the number of *l*-mers that are unique in an individual genome but are not unique in the metagenome is small. We compute this value for pairs of genomes separated by different taxonomic distances. Figure 3 shows the density function of the fraction of *l*-mers that lost their uniqueness due to common repeats, *i.e.*$1-n^{\mathrm{uniq}}/(n_{1}^{\mathrm{uniq}}+n_{2}^{\mathrm{uniq}})$. We can see that the bigger is the phylogenetic distance, the fewer unique *l*-mers are lost.

**Figure 4:**
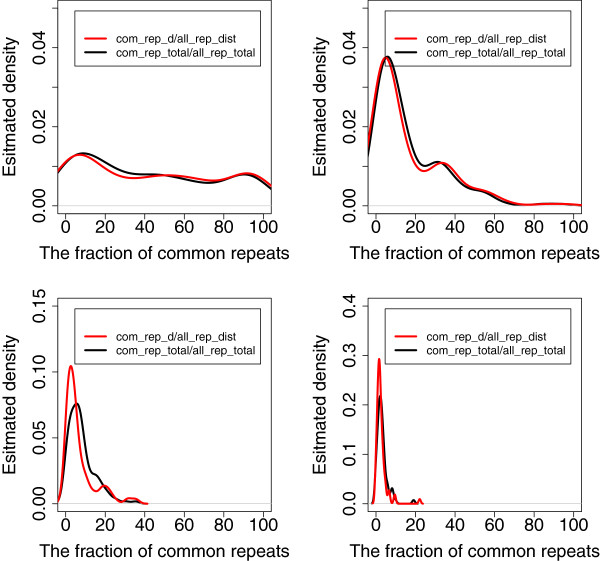
**The fraction of common repeats.** Estimated density function of the ratio of the number of common repeats (or distinct common repeats) to the total number of all distinct repeats (or all repeats, respectively). Top left: pairs of genomes from the same genus but different species. Top right: pairs of genomes from the same family but different genera. Bottom left: pairs of genomes from the same order but different families. Bottom right: pairs of genomes from the same class but different orders.

From now on, by “unique *l*-mers” we will mean *l*-mers that appear only once in all the genomes. The remaining *l*-mers are repeats. We will further classify the repeats into two groups: *individual repeats* are *l*-mers which appear only in one genome (but have several copies) and *common repeats* are *l*-mers that appear in at least two genomes (see Figure 1). Our final observation is: (3) If genomes are separated by sufficient phylogenetic distances (they are at least from different families), then most of the repeats are individual repeats. In addition, the bigger is the phylogenetic distance between genomes, the fewer the common repeats. Figure 4 demonstrates the validity of this observation.

**Figure 5:**
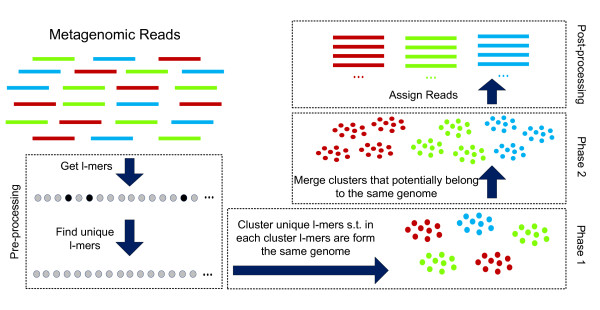
Flowchart of the algorithm.

Our algorithm is based on these three observations. Since most of the *l*-mers are unique in a metagenome, we can cluster the unique *l*-mers by using their common membership in reads so that each cluster contains *l*-mers from only one genome in the first phase. The second phase of our algorithm uses the property that most of repeats are specific to an individual genome. This allows us to merge clusters using the repeated *l*-mers in the metagenome. Figure 5 illustrates a flowchart of our algorithm. Each main step of the algorithm is explained below.

### Finding unique *l*-mers

Before performing the first phase of the algorithm, which clusters the unique *l*-mers, *l*-mers have to be separated into unique *l*-mers and repeats. This is done by choosing a threshold value *K* for the counts of *l*-mers so that *l*-mers with counts less than *K* are most likely unique and the remaining are most likely repeats. Below, we discuss how to chose *K*.

**Figure 6:**
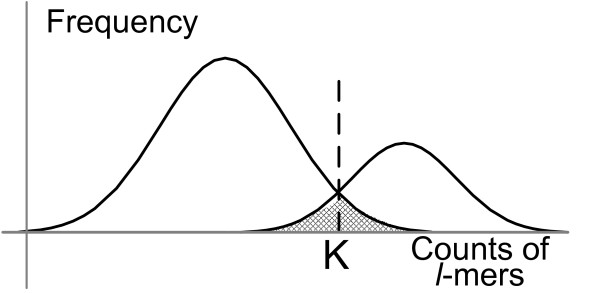
**Threshold choice for the separation of *l*-mers from different distributions.***K* is a threshold to separate *l*-mers from two distributions.

**Figure 7:**
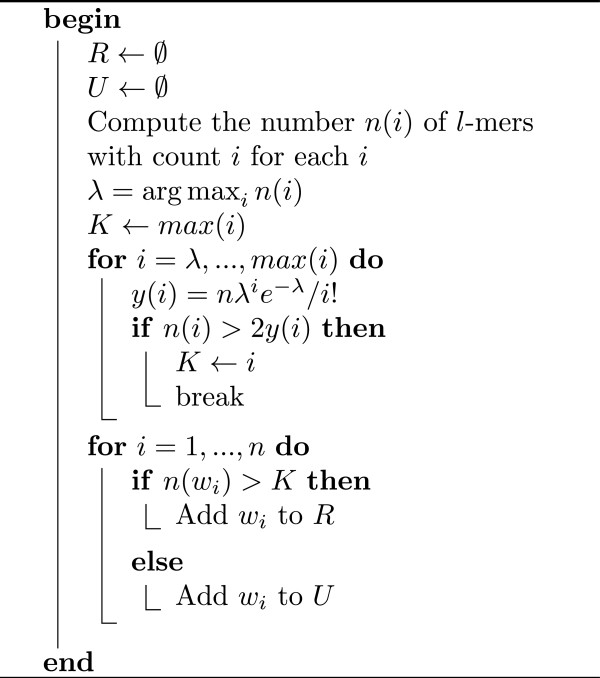
**Algorithm 1.** Detection of unique and repeated *l*-mers. Given *l*-mers *w*_*i*_,*i* = 1,…,*n*, and their counts *n*(*w*_*i*_), the algorithm classifies *l*-mers into repeats *R* and unique *l*-mers *U*.

First, consider error-free metagenomic reads of genomes with equal abundance levels. Let *n* be the number of distinct *l*-mers *w*_1_,*w*_2_,…,*w*_*n*_ with counts *x*(*w*_1_),*x*(*w*_2_),…,*x*(*w*_*n*_). Let *n*(*i*) be the number of distinct *l*-mers with counts *i*. As we discussed in the previous section, the unique *l*-mers follow a Poisson distribution and we may approximate the parameter of the Poisson distribution by the most frequent count of any *l*-mers because most *l*-mers are supposed to be unique. Then, given the estimated parameter, we can estimate the expected number of *l*-mers with counts *i*, *y*(*i*). Figure 6 shows the count distributions of unique and non-unique *l*-mers, where the non-unique *l*-mers (*i.e.*, repeats) are assumed to be from a mixture Poisson distributions and the shaded area shows the expected rate of misclassified *l*-mers for the given threshold value *K*. In the figure, if we choose the threshold higher or lower, more repeats or unique *l*-mers would be undetected, respectively. As a balance, we would choose the intersection point of the two distributions as shown in Figure 6. Although we do not know the distribution of the repeats, we can see that the observed number of *l*-mers with count *K* is twice the expected number of unique *l*-mers with count *K*, and this ratio increases for count values greater than *K*. Based on this intuition, we can estimate the value of *K*. The details are given in Figure 7. A similar approach is used to deal with sequencing errors, by finding a threshold value for counts of *l*-mers that separates unique *l*-mers and *l*-mers with errors.

The set *U* of unique *l*-mers is then used to construct a graph which can help detect more repeats and will be used to do the clustering. The nodes of the graph *G* correspond to the elements of *U* and there is an edge between two nodes if both *l*-mers are contained in a same read. To remove previously undetected repeats, we use the fact that nodes that correspond to truly unique *l*-mers cannot have more than 2(*L* − *l*) neighbors.

### Clustering the unique *l*-mers

**Figure 8:**
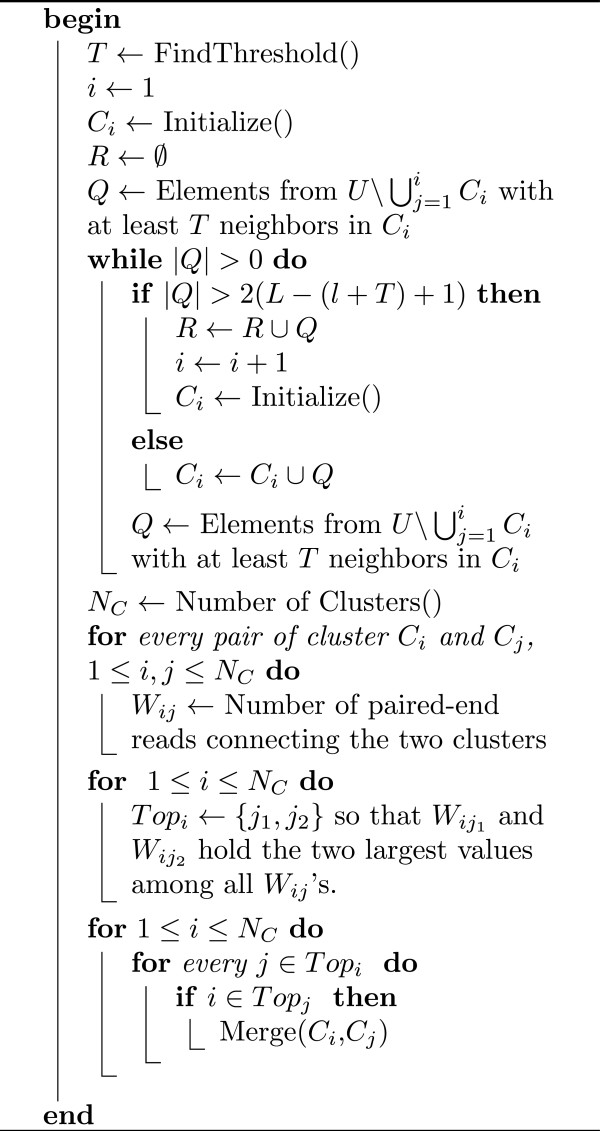
**Algorithm 2.** Clustering of the unique *l*-mers. Given the graph of unique *l*-mers *G* = (*U*,*E*), cluster *l*-mers in *U*.

We use graph *G* described above to perform the clustering. The purpose is to obtain clusters so that each cluster contains unique *l*-mers from only one genome. Note that the number of such clusters for each genome can be large. We initialize the first cluster with the *l*-mers from a randomly selected read and then iteratively find sets of unclustered nodes that are connected to at least *T* nodes in the current cluster (the choice of *T* is discussed later in the subsection). It is important to note that the number of unique *l*-mers we can add at each step is limited by 2(*L* − (*l* + *T*) + 1), since we could add *l*-mers from both ends of a read. If we need to add more than this many *l*-mers at some step, it means that we have encountered true repeats that have not been removed and thus we stop expanding the current cluster. We also stop expanding the current cluster if no more nodes could be added. Then we go to the next iteration and construct the next cluster. For each such subsequent iteration, we initialize a new cluster with *l*-mers from some read that does not correspond to any of the current clusters. A read corresponds to a cluster if at least a half of its *l*-mers belong to the particular cluster. We create new clusters until there are no more unclustered reads left. At the end of clustering, we obtain a set of disjoint clusters of *l*-mers. The paired-end information is then used to consolidate the clusters. The details are given in Figure 8.

Threshold *T* (the minimum required number of edges between an unclustered node and the nodes in a cluster so that the node can be added to this cluster) is chosen to make the expected number of coverage gaps less than one. Recall that the effective coverage is *Cov* = 2*N*(*L* − (*l* + *T*) + 1)/(*G* − *L* + 1) and expected number of gaps is 2*Ne*^−*Cov*^[28].

### Merging clusters and the final clustering of metagenomic reads

**Figure 9:**
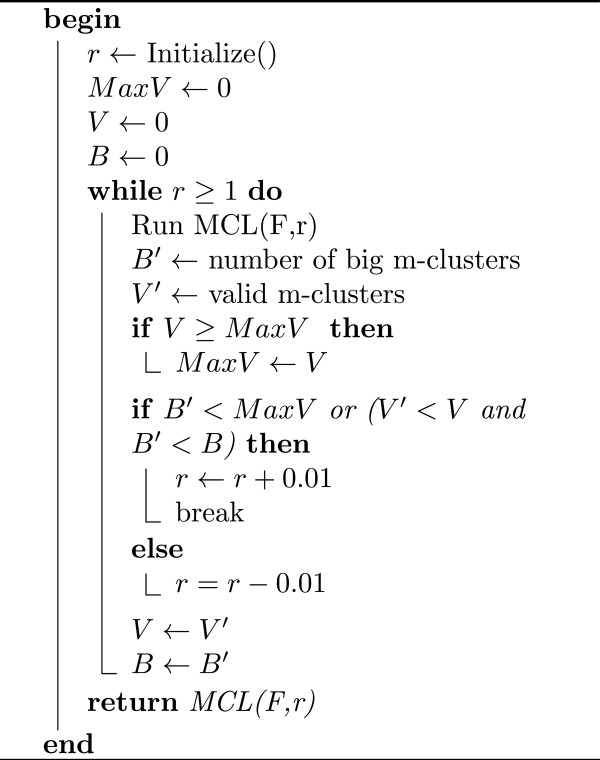
**Algorithm 3.** Merging clusters. Given the weighted graph *F* = (*C*,*W*), construct the m-clusters.

The goal of the second phase is to merge clusters obtained during the previous phase, based on the repeats and information provided by the paired-end reads. First, for each cluster *C*_*i*_, we compute the set of repeats *R*_*i*_that may potentially belong to the same genome as the unique *l*-mers in *C*_*i*_. Each *R*_*i*_consists of two types of *l*-mers. For each read corresponding to cluster *C*_*i*_, it may contain some number of repeats. These repeated *l*-mers are assigned to the set *R*_*i*_. For each read corresponding to *C*_*i*_, we also consider its mate (in a paired-end read) and add to *R*_*i*_all *l*-mers of the mate that have not been assigned to any clusters. Then for each pair of sets *R*_*i*_ and *R*_*j*_, we find the intersection of these sets, *R*_*ij*_. Then, we build a weighted graph *F*, where nodes correspond to clusters *C*_*i*_ and the weight of an edge (*ij*) equals the size of set *R*_*ij*_. Finally, the clusters are merged by using the algorithm MCL [30] on the graph *F*. MCL is an efficient algorithm for clustering sparse weighted graphs and ideal for our situation. To avoid confusion, we will call clusters produced by MCL the *m-clusters*. MCL has a parameter (we denote it by *r*), corresponding to granularity of clusters. We use an iterative algorithm to find the best parameter so that the m-clusters are big enough (in terms of the number of *l*-mers contained in each m-cluster) and the total weight of connections between elements within an m-cluster is higher than the total weight of connections between two different m-clusters. Let us call m-clusters that satisfy the first property *big*, and a subset of big m-clusters that satisfy the second property (with respect to all other big m-clusters) *valid*. We start with a parameter *r* which corresponds to a high granularity and evaluate the resultant clusters in terms of size and validity. Based on the evaluation, we either decrease the parameter to have less granularity or choose the current value of *r* as the parameter for MCL. We obtain final clusters of the unique *l*-mers by merging clusters that belong to the same m-cluster (see Figure 9 in for details).

Now we discuss how to define big and valid m-clusters. The minimum size of a big m-cluster is specified by the user based on the minimum expected length of a genome. Valid m-clusters are chosen from big m-clusters in the following way. Let *W*_*jj*_and *W*_*ii*_be the total weights of the connections within each of the m-clusters *j* and *i*, and *W*_*ij*_ the total weight of the connections between these two m-clusters. The big m-cluster *i* is defined to be valid if for every other big m-clusters *j*, the inequality $\sqrt{\frac{W_{\mathrm{ij}}}{\mathrm{WiiWjj}}}>10^{-3}$ holds. The threshold of 10^−3^ is chosen empirically.

In the final step of the algorithm, the reads are assigned to the resultant clusters of unique *l*-mers. Iterative algorithm is used to assign the reads. At the first step, each reads that correspond to some cluster is assigned to this cluster. During the second step, unassigned reads that have assigned mates are assigned to the same clusters as their mates. In the third step, for each cluster of unique *l*-mers we add all the *l*-mers from the reads assigned to the cluster. We iteratively repeat the three steps for the unassigned reads until no more reads can be assigned. If the read correspond to several clusters, we assign it to one of the clusters.

### Handling genomes with arbitrary abundance levels

We would like to extend the above algorithm to metagenomic data containing genomes with different abundance levels. If the abundance level difference is not significant, the above algorithm would still work well. In this case, the number of wrongly determined unique *l*-mers and repeats in the first phase of the algorithm may slightly increase, but the clustering of *l*-mers based on their counts using the Poisson mixture model may incur a significantly higher drop of performance. For genomes with significantly different abundance levels, it makes sense to first separate reads according to genome abundance levels. Otherwise, repeats from genomes with lower abundance levels will not be detected, which could lead to a significant increase of granularity in the clustering result produced by the first phase of the above algorithm. For this reason, we propose to use the algorithm AbundanceBin [23] for the initial abundance-based binning of reads. Then we run the first phase of our method for each of the subsets of reads. For the second phase, we use all the reads to find the connections between clusters so that connections between clusters from genomes with low abundance levels are properly recovered, but MCL is performed on each subset separately.

A key question is what ratios of abundance levels should be considered as significant? This ratio depends on the actual values of abundance levels and also on the sizes of the genomes. Given abundance levels *λ*_1_ and *λ*_2_(*λ*_1_ < *λ*_2_), genome sizes *G*_1_ and *G*_2_, and a threshold *K* for classifying *l*-mers into the two genomes based on count frequencies, we can estimate the expected rate of misclassified *l*-mers from the count distributions of the *l*-mers in these two genomes as discussed in the “Methods” section. More specifically, the shaded area in Figure 6 represents the expected fraction of misclassification for two distributions. The number of *l*-mer in this area is $l_{2}\overset{K-1}{\underset{i=1}{\sum }}\frac{\lambda_{2}^{i}e^{-\lambda_{2}}}{i!}+l_{1}\overset{\text{Max}}{\underset{i=K}{\sum }}\frac{\lambda_{1}^{i}e^{-\lambda_{1}}}{i!}$. So, we first use AbundanceBin to predict the parameters of count distributions (*i.e.*, the abundance ratios and genome sizes) and then compute the expected rate of misclassification. If this rate is unacceptable (we used 3% as the threshold in the experiments), it means that the abundance levels are not significantly different and thus we do not run AbundanceBin.

## Experimental results

We test the performance of our algorithm on a variety of synthetic datasets with different numbers of species, phylogenetic distances between species, abundance ratios and sequencing error rates. Although simulated datasets do not capture all characteristics of real metagenomic data, there are no real benchmark datasets for NGS metagenomic projects and thus they are the only available option. Also, to the best of our knowledge, there are no algorithms that are designed specifically for separating short NGS reads from different genomes. Although similarity-based methods work on short reads, they explore the taxonomic content of metagenomic data according to known genomes rather than classifying reads. AbundanceBin classifies reads, but it does not separate genomes with similar abundance levels. Therefore, we modify a well-known genome assembly software, Velvet [26], to make it behave like a genome separation tool and compare our clustering results with those of the modified Velvet. In addition, we compare the performance of our algorithm with the well-known composition-based method CompostBin [17] on simulated metagenomic Sanger reads. We also apply the algorithm to a real metagenomic dataset obtained from gut bacteriocytes of the glassy-winged sharpshooter and achieve results consistent with the original study [31].

### Simulated data sets

We use MetaSim [32] to simulate paired-end Illumina reads for various bacterial genomes to form metagenomic datasets. MetaSim is a software for generating metagenomic datasets with controllable parameters, such as the abundance level of each genome, read length, sequencing error rate and distribution of errors. Thus, it can be used to simulate different sequencing technologies and generate reads from available completely sequenced genomes (for example, those in the NCBI database). In our experiments, paired-end reads of length 80 bps are considered, with the mean insert size 500 bps and deviation 20 bps. The number of reads for each experiment is adjusted to produce sufficient coverage depth (ranging between 15 and 30). The sequencing error model is set according to the error profile of 80 bps Illumina reads. A detailed description of MetaSim parameters is provided in Additional file 1.

The first experiment is designed to test the performance of our method on a large number of datasets of varying phylogenetic distances. For this experiment, we create 182 synthetic datasets of 4 categories. Each dataset of the first category contains genomes from the same genus but different species. Datasets in the second category consist of genomes from the same family but different genera, datasets in the third category involve genomes from the same order but different families, and datasets in the fourth category involve genomes from the same class but different orders. Genomes in each test are randomly chosen according to a category of phylogenetic distances and assumed to have the same abundance levels. The number of genomes in the datasets varies from 2 to 10 and depends on the number of available complete sequences for each taxonomic group and on the level of the group. Tests on genomes from the same genus typically involve 2 to 4 genomes since such genomes are similar to each other and hard to separate, while tests on genomes from the same class may involve up to 10 genomes. Totally, we have 79 experiments concerning a genus, 66 concerning a family, 29 concerning an order, and 8 concerning a class. These datasets involve 515 complete genomes from the NCBI.

We also performed some small-scale experiments to test the performance on genomes with different abundance levels and on reads with sequencing errors. For each of the experiments, we choose 10 random sets of genomes from the 182 datasets. For each set of genomes, two metagenomic dataset are simulated, one with abundance ratio 1:2 and and the second with the error model but abundance ratio 1:1. Finally, we test the performance of the combination of our algorithm and AbundanceBin on a dataset of 4 genomes with abundances 1:1:4:4. The exact species combinations used in all simulated datasets are listed in Additional file 2.

### Comparison with modified velvet

Due to the lack of methods for separating short NGS reads into genomes, we modify a well-known genome assembler, Velvet [26], so it behaves like a genome separator. Genome assemblers such as Velvet often work with metagenomic data and produce contigs that may actually correspond to sections of individual genomes. Hence, we run Velvet to obtain a set of contigs and use each contig to define a cluster of *l*-mers. This is equivalent to the first phase of our algorithm. The only difference is that all *l*-mers (instead of unique *l*-mers) are clustered. For each read contained in a cluster, we add the *l*-mers in the mate of the read to the cluster, and then construct a weighted graph whose nodes represent clusters and edges are weighted by the number of common *l*-mers shared by the clusters connected by each edge. Finally, we apply our merging algorithm to the constructed graph. Based on a series of experiments with the Velvet parameters, we chose *l*-mer length as 31, which results in the highest N50 in most of the experiments. We also set the coverage cutoff to half the coverage (*i.e.*, abundance level) of the least abundant genome in the dataset, so that Velvet can deal with genomes with different abundance levels without filtering out low coverage contigs.

### Performance evaluation

To evaluate the results of clustering, there are a number of factors that should be considered. First of all, we would like most of the reads from each genome to be located in one cluster. In other words, each genome should correspond to a unique cluster that contains most of its reads. We say that a genome has been *broken* if there is no cluster that contains more than a half of all its reads. It may happen that several genomes correspond to the same cluster. In this case, we assign the cluster to all the genomes, and say that the genomes are not separated. We will measure the performance of our algorithm in terms of pairwise separability. For example, if a dataset contains 5 genomes, where 3 of them are located in one cluster, and each of the other two are located in its own cluster, then in the pairwise evaluation, we consider the separability of all 10 pairs of genomes. Since 3 pairs of genomes are not separated while the other 7 are separated, the separability rate is 70%. During the separability analysis, we remove broken genomes from consideration. Besides separability, we are interested in the precision and sensitivity of our algorithm on the separated genomes. Since we assign a genome to the cluster that has most of its reads, it is also interesting to know how many of its reads are wrongly assigned to other clusters. We call this *sensitivity*. One way to estimate sensitivity is to compute how many reads are correctly assigned to each cluster and divide it by the total number of reads that should be in this cluster. Here, true positives are the reads from all genomes located in this cluster. However, consider the case when we have two genomes in a cluster, of lengths 1 Mbps and 5 Mbps respectively. Then, even if sensitivity is very low for the first genome, the overall sensitivity (for all genomes in the cluster) will not be significantly affected. Another way to normalize sensitivity is by computing sensitivity for each genome in the cluster separately and then to find the average of these sensitivities. We use the second approach. To compute *precision* of a cluster, we find the ratio of the reads that are wrongly assigned to the cluster to the total number of reads in the cluster.

To summarize the results for a set of experiments, we compute separability based on the total number of pairs of genomes in all the experiments. For the precision and sensitivity, we take the average values for all the clusters from all the experiments.

### Experiments on genomes separated by different phylogenetic distances

**Figure 10:**
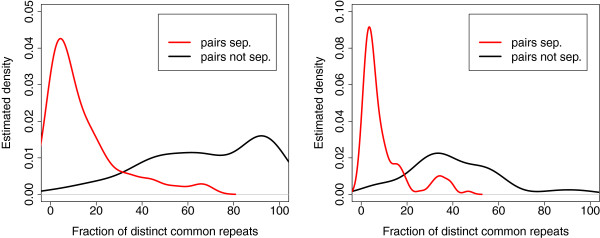
**The fraction of distinct common repeats for separated and unseparated genomes.** Estimated density function of the ratio of the number of distinct common repeats to the number of distinct repeats. Left: pair of genomes from the same genus but different species. Right: pairs of genomes from the same family but different genera.

Our experimental results on metagenomic datasets containing genomes with different phylogenetic distances are summarized in Table 1. For genomes from the same genus, separability rate is 45%. It increases to 77% for genomes from the same family and more than 97% for higher level taxonomic categories. For separated pairs of genomes, our sensitivity increases from 90% for genomes from the same genus to 95% for genomes from the same order. The range of precision is from 95% to 98%. These results are consistent with our expectation for correlation between separability and phylogenetic distance. Figure 10 shows the estimated density functions of the fraction of common repeats for separated and unseparated pairs of genomes at the genus and family levels. It confirms our conjecture that the higher is the faction of common repeats, the harder is separation and the worse is the accuracy of our method. Table 1 also shows the performance of the Velvet-based approach. Its separability rate is lower than ours (32% for genus, 62% for family and 91% for order). Its sensitivity and precision numbers are also worse than ours at the genus and family levels but become slightly higher at the order level.

**Table 1:** Performance of our method and the Velvet-based approach on pairs of genomes with different phylogenetic distances

		**# of genomes**	**# of pairs**	**Broken**	**Separated**	**Sensitivity**		**Precision**	
						**Mean**	**Stdv**	**Mean**	**Stdv**
Species	TOSS	229	184	3	83	90.38	8.71	95.55	5.96
	Velvet	229	156	22	51	84.11	12.70	92.25	7.88
Genus	TOSS	171	147	2	113	93.40	9.39	97.07	5.52
	Velvet	171	123	15	77	89.96	12.97	94.95	8.45
Family	TOSS	80	79	2	75	94.98	6.56	97.46	5.86
	Velvet	80	78	2	71	91.90	8.90	97.25	4.16
Order	TOSS	35	71	0	70	95.14	4.87	97.79	2.49
	Velvet	35	71	0	69	95.79	4.17	98.77	1.69

### Handling sequencing errors

Our approach for handling sequencing errors is very simple: we filter out *l*-mers with counts lower than a certain threshold, since these infrequent *l*-mers are likely to contain errors. However, there is a simple intuition behind it. We can aggressively remove potential errors without attempting to correct them or being afraid to lose important information, assuming that the genomes are sufficiently covered by the reads. Note that we could be more aggressive than genome assemblers in throwing out infrequent *l*-mers here because (i) when the genomes are sufficiently covered, the filtration will not lead to many more gaps, and (ii) we are less concerned with the fragmentation of genomes.

In Table 2, we summarize our experimental results on pairs of genomes with and without sequencing errors. We can see that our method is able to separate more pairs of genomes when the reads are error-free. However, when broken genomes are discounted, the method actually achieves a slightly higher sensitivity and precision on data with errors. The Velvet-based method has a slightly worse performance, it separates fewer pairs of genomes and achieves a lower sensitivity and precision on both data with and without errors.

**Table 2:** Performance of the method on data with and without sequencing errors

		**# of genomes**	**# of pairs**	**Broken**	**Separated**	**Sensitivity**	**Precision**
TOSS	With errors	24	15	2	15	96.84	98.03
	Error-free	24	18	0	18	93.48	96.08
Velvet	With errors	24	16	1	14	87.32	96.84
	Error-free	24	18	0	17	88.24	95.06

### The issue of abundance levels

In this section, we analyze the ability of our method to separate genomes with different abundance levels. First, we test our algorithm (without any modification) on pairs of genomes with abundance ratio 1:2 and compare the results with those on the same set of pairs of genomes but with identical abundance levels. The results are summarized in the Table 3. We can see that sensitivity slightly drops on genomes with different abundance levels, but precision actually improves a little. On the other hand, separability of the Velvet-based method drops significantly.

**Table 3:** Performance on synthetic datasets with abundance ratio 1:2

		**# of genomes**	**# of pairs**	**Broken**	**Separated**	**Sensitivity**	**Precision**
TOSS	DiffAbund	24	18	0	17	91.00	98.25
	IdentAbund	24	18	0	18	93.48	96.08
Velvet	DiffAbund	24	16	1	13	91.88	97.48
	IdentAbund	24	18	0	17	88.24	95.06

We also test the performance of a combination of AbundanceBin and our algorithm on a set of four genomes with abundance levels (1:1:4:4) and compare its result with that of our (original) algorithm on the same set of genomes with identical abundance levels. The results are summarized in Table 4. As we can see, the result on data with varying abundance levels is actually better. Sensitivity and precision increase from 92% and 93% on data with identical abundance levels to 97% and 99% on data with varying abundance levels. In order to explain this (somewhat counter-intuitive) phenomenon, we analyzed intermediate results, and found that two of the six pairs of genomes, (1,3) and (2,4), have high percentages of common repeats. These common repeats negatively affected the result on data with identical abundance levels. However, they did not cause any trouble for the test on data with varying abundance levels since for both pairs, reads from different genomes were separated by AbundanceBin early on due to the difference in their abundance levels. On the other hand, the precision of the Velvet-based method drops significantly.

**Table 4:** Performance on synthetic datasets with abundance ratio (1:1:4:4)

		**# of genomes**	**# of pairs**	**Broken**	**Separated**	**Sensitivity**	**Precision**
TOSS	DiffAbund	4	6	0	6	97.42	99.81
	IdentAbund	4	6	0	6	92.10	93.81
Velvet	DiffAbund	4	6	0	5	91.41	85.24
	IdentAbund	4	6	0	6	90.44	92.65

### Comparison with compostBin

In this section, we compare the performance of our algorithm with a composition-based binning algorithm, CompostBin [17]. Note that composition-based methods require sufficiently long reads while TOSS is designed to separate short NGS reads. On the other hand, our method requires a high coverage depth. To compare the performance with CompostBin, we use the simulated paired-end Sanger reads of length 1000 bps provided in [17]. We slightly adapt our method to handle longer reads and lower coverage. In particular, we use a higher threshold in the prediction of unique *l*-mers. Also, we cut the Sanger reads into fragments of length 80 bps before constructing the graph of unique *l*-mers in order to minimize memory usage. The linkage information of the fragments belonging to a same read will be recovered and taken advantage of later in the cluster merging phase. Normalized error rates (as defined in [17]) for our algorithm and for CompostBin are reported in Table 5. Note that in the last three datasets, the average coverage of genomes with lower abundance levels (not shown in the table) is close to 1 and, therefore, is insufficient for our algorithm. In addition, we simulate Illumina reads from the same sets of genomes with a coverage depth between 15 and 30. Normalized error rates for these datasets are shown in the last column of Table 5. The highest error rates of our algorithm on Sanger and Illumina reads are 4.74% and 4.92% respectively, and are less than 10% for CompostBin. For some of the Sanger datasets, the performance of our algorithm is slightly worse compared to CompostBin and for the others, it is slightly better. The performance of our algorithm on the corresponding Illumina datasets is better in most of the cases. Clearly, the higher coverage depths in Illumina datasets helped. A high coverage depth is essential for the accurate prediction of unique and repeated *l*-mers in the preprocessing phase of our algorithm.

**Table 5:** Comparison with CompostBin on the datasets described in [17]

**Species**	**Ratio**	**Phylogenetic**	**CompostBin’s**	**TOSS (Sanger)**	**TOSS (Illumina)**
		**distance**	**error**	**error**	**error**
*Bacillus halodurans* &*Bacillus subtilis*	1:1	Species	6.48	1.05	1.38
*Gluconobacter oxydans*&	1:1	Genus	3.39	4.72	4.92
*Granulibacter bethesdensis*					
*Escherichia coli* &*Yersinia pestis*	1:1	Genus	10.00	3.11	2.58
*Methanocaldococcus jannaschii* &	1:1	Family	0.51	0.22	0.01
*Methanococcus maripaludis*					
*Pyrobaculum aerophilum* &	1:1	Family	0.28	1.05	0.01
*Thermofilum pendens*					
*Gluconobacter oxydans* &	1:1	Order	0.98	4.74	0.01
*Rhodospirillum rubrum*					
*Gluconobacter oxydans,*	1:1:8	Family and Order	7.7	-	6.45
*Granulibacter bethesdensis* &					
*Nitrobacter hamburgensis*					
*Escherichia coli, Pseudomonas putida* &	1:1:8	Order and	1.96	-	0.15
*Bacillus anthracis*		Phylum			
*Escherichia coli, Pseudomonas putida,*	1:1:	Species, Order,	4.52	-	0.80
*Thermofilum pendens,*	1:1:	Family,			
*Pyrobaculum aerophilum,*	2:14	Phylum, and			
*Bacillus anthracis* &*Bacillus subtilis*		Kingdom			

Note that some datasets involve genomes separated at several different taxonomic levels.

### Performance on a real dataset

A metagenomic dataset obtained from gut bacteriocytes of the glassy-winged sharpshooter, *Homalodisca coagulata*, is known to consist of (Sanger) reads from *Baumannia cicadellinicola*, *Sulcia muelleri* and some miscellaneous unclassified reads [31] and studied in [17]. We apply our algorithm, adapted to handle Sanger reads as discussed in the previous section, to the dataset. As in [17], we only measure our ability to separate the reads from *Baumannia cicadellinicola* and *Sulcia muelleri*. The sensitivity of the classification achieved by our algorithm is 92.21% and the normalized error rate is 1.59%, which is lower than the normalized error rate of 9.04% achieved by CompostBin as reported in [17].

## Conclusions and Discussions

While the NGS sequencing technologies are very promising for metagenomic projects due to their great sequencing depths and low costs, they also present new challenges in the analysis of metagenomic data because of their short read lengths. In this paper, we developed an algorithm for separating short paired-end NGS reads from different bacterial genomes of similar abundance levels and then combined the algorithm with AbundanceBin [23] to handle arbitrary abundance ratios. We have shown that our algorithm is able to separate genomes when the number of common repeats is small compared to the number of genome-specific repeats. Since the fraction of common repeats in genomes correlates with the phylogenetic distance between the genomes, it is hard to separate genomes of closely related species. However, for the genomes that are separated by sufficient phylogenetic distance, they share few *l*-mers and can be separated with high precision and sensitivity.

Our algorithm called TOSS was coded in C. Its running time and memory requirement depend on the total length of all the genomes present in a metagenomic dataset and on the number of reads. The first phase of the algorithm is the most time and memory consuming. In this phase, a graph of *l*-mers is constructed and the clustering of unique *l*-mers is performed. The size of the graph is proportional to the total size of the genomes and 0.5 GB of RAM is required for every million bases of the genomes. In the experiments, we ran the algorithm on a single CPU with 2.8GHz AMD machine and 64GB RAM. Each of the small-scale tests involving 2-4 genomes of total length of 2-6 Mbps was completed within 1-3 hours and required 2-4 GB of RAM. A test on 15 genomes with the total length of 40 Mbps ran for 14 hours and required 20GB of RAM.

Our algorithm can be further improved. In this paper, to separate the input reads, we construct a graph by using the information about *l*-mers from all the reads. After clustering the unique *l*-mers, some clusters are merged if they potentially belong to the same genome. To find connections between clusters, paired-end reads and common repeats are used. However, we believe that additional information can be used to improve the algorithm’s ability in predicting whether two clusters potentially belong to the same genome. For example, the compositional properties of the clusters of unique *l*-mers may be used to complement the repeat-based information.

In future work, we plan to explore the compositional properties of the clusters of unique *l*-mers and try to improve the performance of our algorithm by combining the compositional properties with the distribution of *l*-mers in reads.

## Endnote

This is a *WABI’2011* special issue invited paper.

## Competing interests

The authors declare that they have no competing interests.

## Author’s contributions

OT and TJ conceived the framework of the algorithm. OT implemented and tested the algorithm. OT wrote the manuscript with some help from TJ and JB. All authors read and approved the manuscript.

## Supplementary Material

Additional file 1 MetaSim Parameters. Detailed description of MetaSim parameters used to create the simulated datasets. Click here for file

Additional file 2 Simulated Datasets. The exact species combinations used in all simulated datasets. Click here for file
